# Clinical characteristics, molecular features and *in vitro* susceptibility to sulbactam-durlobactam, cefiderocol, and eravacycline in NDM-1-producing versus OXA-23-producing carbapenem-resistant *Acinetobacter baumannii* isolates from children in Guangxi, China

**DOI:** 10.3389/fcimb.2026.1965770

**Published:** 2026-10-08

**Authors:** Minxue Liu, Mengjun Li, Chenglan Yan, Liying Huang, Huan Zhang

**Affiliations:** Department of Laboratory Medicine, Children’s Hospital, Maternal and Child Health Hospital of Guangxi Zhuang Autonomous Region, Nanning, China

**Keywords:** carbapenem-resistant *Acinetobacter baumannii*, drug resistance, NDM metallo-β-lactamase, pediatric patients, whole-genome sequencing

## Abstract

**Background:**

Carbapenem-resistant *A. baumannii* (CRAB) has been recognized as a critical priority pathogen responsible for a wide range of hospital-acquired infections.

**Methods:**

We included pediatric CRAB infection cases at a Maternal and Child Health Hospital in Guangxi, China, from January 2019 to December 2024. The bacterial isolates had previously undergone whole-genome sequencing (WGS). An NDM-1-producing CRAB strain isolated from a bloodstream infection was subjected to Nanopore sequencing to characterize the NDM-1-encoding plasmid. We then retrospectively analyzed the risk factors for NDM-1-producing CRAB infection in children. In addition, the NDM-1-producing and OXA-23-producing CRAB strains were subjected to antimicrobial susceptibility testing (AST) against sulbactam-durlobactam, cefiderocol, and eravacycline using the micro-broth dilution method, and their susceptibility profiles were compared. Associated resistance genes were analyzed.

**Results:**

A total of 131 pediatric patients with CRAB infections were included in this study. Based on the WGS data, 33 CRAB strains harboring *bla*_NDM-1_ were classified into the NDM-1-producing group, while 98 CRAB strains harboring *bla*_OXA-23_ were classified into the OXA-23-producing group. On multivariable analysis, indwelling urethral catheters (OR = 0.293; *P* = 0.009) and white blood cell (WBC) count (OR = 0.896; *P* = 0.039) were negatively associated with NDM-1-producing CRAB infection, yet positively associated with OXA-23-producing infections (*P* < 0.05). WGS analysis revealed that 32 of the 33 (97.0%) NDM-1-producing CRAB isolates co-carried *bla*_PER-1_, which has been reported to be associated with cefiderocol resistance in *A. baumannii*. Plasmid mapping showed that the same plasmid co-harbored *bla*_NDM-1_ and *bla*_PER-1_, together with a type IV secretion system (T4SS). The resistance rate of NDM-1-producing CRAB isolates to sulbactam-durlobactam was 100%, and to cefiderocol was 66.7%—both rates being significantly higher than those observed in the OXA-23-producing group (1.0% and 0.0%, respectively; *P* < 0.001). However, all 131 isolates demonstrated susceptibility to eravacycline *in vitro*.

**Conclusion:**

Clinical characteristics and antibiotic susceptibility differed significantly between NDM-1-producing and OXA-23-producing CRAB infections. Carbapenemase surveillance is critical for the management of CRAB infections among pediatric patients in Guangxi, China, and AST is essential to guide treatment.

## Introduction

*Acinetobacter baumannii* is a significant pathogen that causes various hospital-acquired infections. The clinical threat of *A. baumannii* largely stems from its ability to rapidly develop resistance to various antibiotics, including carbapenems. CRAB is now widespread globally, imposing a heavy burden on patients through elevated mortality, extended hospitalization, and increased healthcare expenditures (Boutzoukas and Doi, 2025). In 2024, the World Health Organization (WHO) has listed CRAB as a critical priority pathogen (Sati et al., 2025). Surveillance data from the China Antimicrobial Surveillance Network (CHINET; https://www.chinets.com) indicate that the rate of CRAB in China has remained at approximately 70% in recent years. Consequently, the clinical management and treatment of CRAB infection pose a considerable challenge in China.

The main resistance mechanism of CRAB is the production of carbapenemases, with OXA-23 being the most common carbapenemase among CRAB isolates (Müller et al., 2023). However, NDM-1 has been increasingly detected in CRAB. In our previous study, NDM-1-producing isolates accounted for 25% of CRAB in pediatric patients at the Maternal and Child Health Hospital of Guangxi (Liu et al., 2026). Sulbactam-durlobactam, approved in China in 2024 (www.nmpa.gov.cn), is recommended by the Infectious Diseases Society of America (IDSA) as a preferred carbapenem-combination regimen, as stated in the 2024 guidance (Tamma et al., 2024) and reaffirmed in the 2026 update (www.idsociety.org/practice-guideline/amr-guidance/). Cefiderocol, which has demonstrated potent *in vitro* activity against CRAB (Abdul-Mutakabbir et al., 2020), is recommended by the IDSA as an alternative combination antibiotic and was approved in China in 2026. Eravacycline has also been approved in China, exhibiting strong *in vitro* activity against multidrug-resistant (MDR) bacteria, including CRAB (Alosaimy et al., 2020). However, the clinical characteristics and *in vitro* susceptibility of these novel antibiotics against NDM-1- and OXA-23-producing CRAB in pediatric patients remain poorly understood.

In this study, we compared the clinical characteristics and antimicrobial susceptibility of sulbactam-durlobactam, cefiderocol, and eravacycline between NDM-1-producing and OXA-23-producing CRAB isolates collected from Guangxi pediatric patients between 2019 and 2024. These efforts aim to assist in clinical surveillance and guide the selection of antibiotics for the treatment of CRAB infection.

## Materials and methods

### Study setting and patients

We enrolled pediatric patients diagnosed with CRAB infection at the Maternal and Child Health Hospital of Guangxi, China, from January 2019 to December 2024. CRAB infection were defined as follows (Bartal et al., 2022): (i) presence of infectious symptoms such as fever, cough, and sputum production; (ii) elevated laboratory inflammatory markers (e.g., leukocytosis, elevated C-reactive protein [CRP], and raised serum procalcitonin [PCT]); and/or (iii) radiographic evidence suggesting infection; (iv) microbiological evidence was established by the recovery of CRAB from a sterile-site culture or the observation of gram-negative bacilli phagocytosed by neutrophils on direct microscopy. Sputum samples were considered acceptable only if they met the following cytological criteria: >25 leukocytes and <5 squamous cells per low-power field. Patients were enrolled if they met the following criteria: (i) CRAB infection confirmed by two senior physicians; (ii) age ≤ 14 years; (iii) availability of complete clinical data; and (iv) preservation of viable clinical isolates. The following were excluded: (i) outpatients, and (ii) subsequent CRAB isolates from patients already included in the study.

### Bacterial isolate identification

A total of 131 non-duplicate clinical isolates of CRAB were included (**Figure 1**). Species identification was performed for all isolates using MALDI-TOF MS (Antobio MS600, Zhengzhou, China). Carbapenem resistance was determined using AST according to the Clinical and Laboratory Standards Institute (CLSI) M100 guidelines (36th edition). All strains were preserved at –80 °C for subsequent analysis.

**Figure 1:**
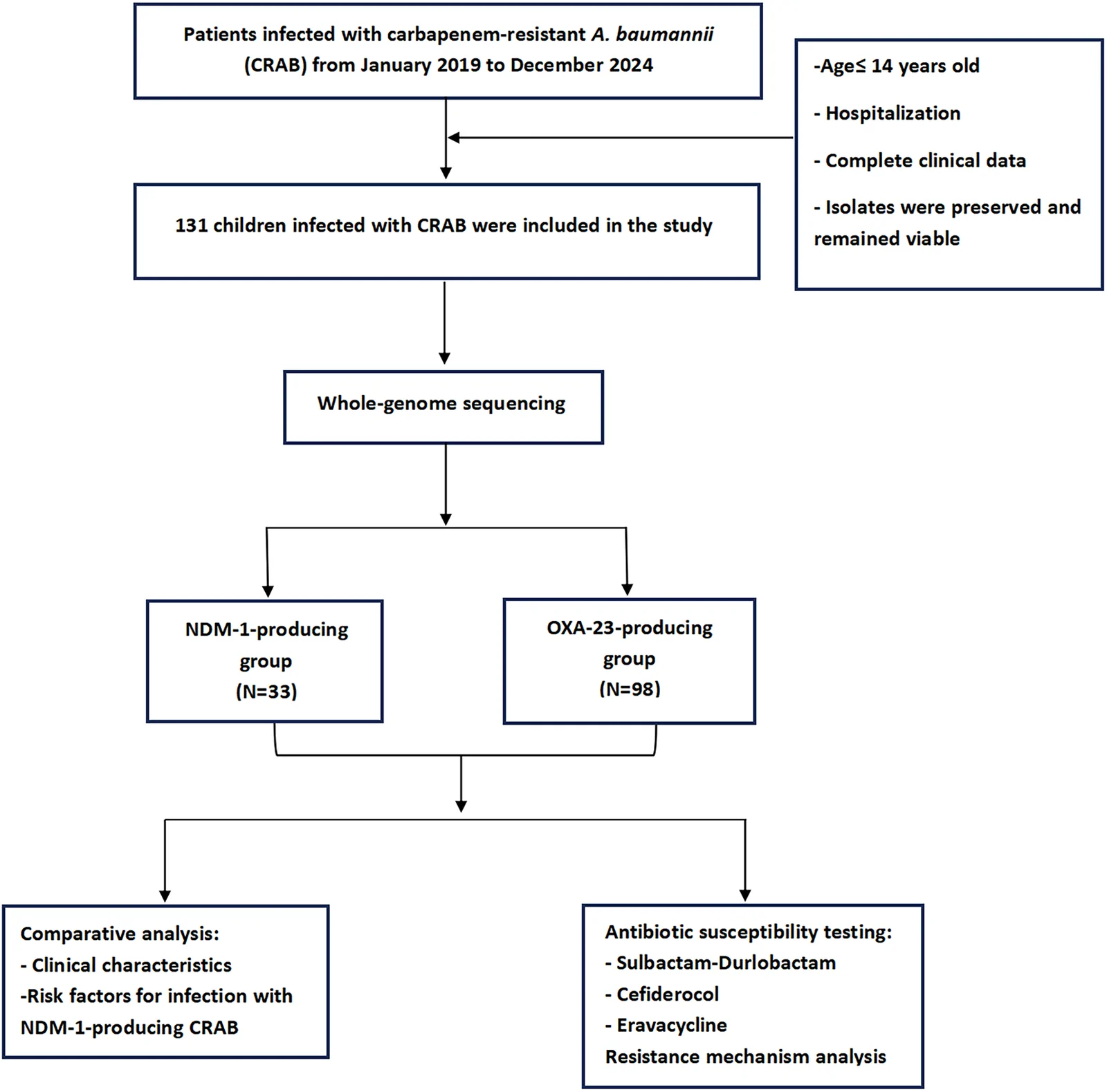
Flowchart of the experimental design.

### Whole-genome sequencing and patient subgrouping

All 131 CRAB isolates were subjected to WGS on the MGI DNBSEQ-T7 (MGI Tech, Shenzhen, China) platform at Sangon Biotech (Shanghai, China). The protocol for the sequencing method was consistent with previous descriptions (Liu et al., 2026). Antimicrobial resistance (AMR) genes in the assembled *A. baumannii* genomes were predicted using the ResFinder 4.4.2 webserver (thresholds: ≥95% identity, ≥60% length; http://genepi.food.dtu.dk/resfinder) and the Resistance Gene Identifier (RGI) from the Comprehensive Antibiotic Resistance Database (CARD; https://card.mcmaster.ca/). Isolates positive for the NDM-1 carbapenemase gene were assigned to the NDM-1-producing group, while those positive for the OXA-23 carbapenemase gene were assigned to the OXA-23-producing group.

### Clinical data collection

We retrospectively queried the hospital’s electronic medical record system to obtain clinical data for all pediatric patients with CRAB infection identified during the study period. The extracted information included demographic characteristics (gender and age), underlying diseases, history of invasive procedures, a history of antibiotic administration, pediatric intensive care unit (PICU) admission, prior hospitalizations, laboratory results within 48 hours of CRAB detection and clinical outcomes.

### Antimicrobial susceptibility testing

AST of the 131 CRAB strains against sulbactam-durlobactam, cefiderocol, and eravacycline was performed according to the CLSI M100 guidelines (36th edition) using the broth microdilution method with commercial panels (Kangtai, Wenzhou, China). Specifically, cefiderocol AST was performed using iron-depleted cation-adjusted Mueller-Hinton broth (ID-CAMHB), as susceptibility to this agent is highly sensitive to iron concentrations. Susceptibility to other commonly used clinical antibiotics—including ceftazidime, ceftriaxone, cefepime, ampicillin-sulbactam, ticarcillin-clavulanic acid, piperacillin-tazobactam, cefoperazone-sulbactam, meropenem, imipenem, amikacin, gentamicin, levofloxacin, ciprofloxacin, sulfamethoxazole, polymyxin B, and minocycline—was determined using the DL-96A system (Dier, Zhuhai, China). Results were interpreted according to the guidelines provided in the CLSI M100 (36th edition). However, as clinical breakpoints for eravacycline against *A. baumannii* are not available from CLSI, susceptibility was interpreted according to the China Committee on Antimicrobial Susceptibility Testing (ChiCAST) guideline, which categorizes isolates with minimum inhibitory concentration (MIC) ≤1 µg/mL as susceptible. *Escherichia coli* ATCC 25922 and *A. baumannii* NCTC 13304 were used as quality control. AST results were compared between the NDM-1- and OXA-23-producing groups.

### Genomic analyses

We identified variants by aligning the assemblies to the reference genome of *A. baumannii* BJAB07104 (GenBank accession: NC_021726.1).

Then, we constructed an single-nucleotide polymorphisms (SNP)-based phylogenetic tree from genome-wide variants identified by Snippy (v 4.6.0) and visualized it using iTOL v6 (https://itol.embl.de/).

We selected an NDM-1-producing CRAB strain GXFY097 isolated from a bloodstream infection for further Nanopore sequencing. Based on Nanopore sequencing, the structure of the NDM-1-encoding plasmid was visualized with Proksee (https://proksee.ca/projects) and subsequently refined with Inkscape. The type IV secretion system (T4SS) gene clusters were predicted using oriTfinder (Li et al., 2018). In order to ensure the NDM-1-encoding plasmid existed in other 32 NDM-1-producing strains, we extracted the complete NDM-1-encoding plasmid sequence from the Nanopore assembly of strain GXFY097 and used it as a reference. The short-read WGS data from the remaining 32 NDM-1-producing strains was then mapped to this plasmid sequence using BWA-MEM v0.7.17.

Sulbactam exerts its antibacterial activity against *A. baumannii* by inhibiting penicillin-binding protein (PBP) 1a, PBP1b, and PBP3. PBP3 mutations, which represent a common resistance mechanism in OXA-23-producing CRAB (Karruli et al., 2023; Xu et al., 2026), were analyzed in sulbactam-durlobactam-resistant OXA-23-producing isolates. The *ftsI* gene, which encodes PBP3, was extracted from the WGS data using a custom bioinformatics pipeline based on BLAST. Subsequently, the *ftsI* gene sequence was translated into an amino acid sequence and compared with the wild-type PBP3 sequence from *A. baumannii* ATCC 19606 using SnapGene v8.0.

In addition to the NDM gene, we also analyzed other major genes associated with increased cefiderocol resistance in *A. baumannii*. We determined the prevalence of PER-1, a class A extended-spectrum β-lactamase (ESBL) previously reported to be associated with cefiderocol resistance in *A. baumannii* (Poirel et al., 2021, Strateva et al., 2026). In addition, cefiderocol resistance is often driven by decreased expression in expression of receptors (including *pir*A and *piu*A) involved in ferric uptake (Asrat et al., 2023; Smoke et al., 2023; Raro et al., 2026; Lai et al., 2026). The iron uptake-associated genes *pir*A and *piu*A were analyzed using the CefiderocolFinder tool (http://github.com/Bryan-vd-Brand/CefiderocolFinder) (van den Brand et al., 2026).

### Statistical analysis

Data were analyzed using IBM SPSS Statistics (version 25.0). If normally distributed, continuous data are shown as the mean ± standard deviation (x̄ ± s); otherwise, they are reported as the median with interquartile range (IQR). Categorical data are expressed as percentages.

We compared continuous variables using the Student’s *t*-test for normally distributed data and the Mann–Whitney U-test for non-normally distributed data. Categorical variables were evaluated with the chi-square test or Fisher’s exact test, as appropriate. A two-tailed *P*-value < 0.05 was considered statistically significant.

To clarify whether there is a high correlation among the factors with *P* < 0.05 in the univariate analysis, we calculated the variance inflation factor (VIF) as part of collinearity diagnostics. Clinically relevant variables significant at *P* < 0.05 in univariate analysis with VIF < 5 were entered into multivariable logistic regression to identify independent risk factors.

## Results

### Clinical characteristics

In total, 131 non-duplicate clinical isolates were recovered from CRAB-infected pediatric patients who presented between January 2019 and December 2024. WGS revealed that 33 strains carried the *bla_N_*_DM-1_ gene and 98 strains carried the *bla*_OXA-23_ gene; however, no strain was found to harbor both genes simultaneously. Therefore, the 131 CRAB isolates were classified into NDM-1-producing (n = 33, 25.2%) and OXA-23-producing (n = 98, 74.8%) groups.

Demographic and clinical data for the pediatric cohort are shown in **Table 1**. Comparisons between the NDM-1-producing group and the OXA-23-producing group revealed no statistically significant differences (*P* > 0.05) in gender, history of prior hospitalizations, underlying diseases, PICU admission, CRAB infection type, or most invasive procedures (mechanical ventilation, central venous catheter [CVC], drainage tube, parenteral nutrition, and nasogastric feeding). Regarding antibiotic exposure, the two groups were largely comparable (*P* > 0.05), with the exception of glycopeptides (*P* = 0.029) and carbapenems (*P* = 0.018). Laboratory parameters, including platelet count (PLT), CRP, albumin (ALB), and prealbumin (PA), also showed no significant differences (*P* > 0.05). The mortality rates among pediatric patients infected with NDM-1-producing and OXA-23-producing CRAB were 30.3% and 38.8%, respectively, with on significant difference (*P* = 0.382).

**Table 1:** Clinical features of children with carbapenem-resistant *Acinetobacter baumannii* infection: NDM-1-producing group vs. OXA-23-producing group.

Factors	Total (n=131),	NDM-1-producing group (n=33)	OXA-23-producing group (n=98)	*P*-value
Basic data				
Gender (male), n (%)	83 (63.4)	18 (54.5)	65 (66.3)	0.224
Age, year, M (IQR)	1 ± 2.5	0.7 ± 1	1 ± 2.7	**0.045**
**Prior hospitalizations**, n (%)	71 (54.2)	16 (48.5)	55 (56.1)	0.446
Underlying diseases, n (%)				
Tumor	4 (3.1)	1 (3.0)	3 (3.1)	0.993
Anemia	66 (50.4)	12 (36.4)	54 (55.1)	0.063
Enteritis	8 (6.1)	1 (3.0)	7 (7.1)	0.679
Congenital heart disease	17 (13.0)	5 (15.2)	12 (12.2)	0.765
Hematonosis	7 (5.3)	1 (3.0)	6 (6.1)	0.679
**PICU**, n (%)	119 (90.8)	32 (97.0)	87 (88.8)	0.293
CRAB infection type, n (%)				
Pneumonia	115 (87.8)	31 (93.9)	84 (85.7)	0.356
Bloodstream infections	8 (6.1)	1 (3.0)	7 (7.1)	0.679
Meningitis	2 (1.5)	0 (0.0)	2 (2.0)	1.000
Abdominal infection	6 (4.6)	1 (3.0)	5 (5.1)	1.000
**Septic shock**, n (%)	23 (17.6)	2 (6.1)	21 (21.4)	**0.045**
Invasive procedures and devices, n (%)				
Mechanical ventilation	117 (89.3)	29 (87.9)	88 (89.8)	0.750
Indwelling urethral catheters	84 (64.1)	14 (42.4)	70 (71.4)	**0.007**
CVC	59 (45.0)	10 (30.3)	49 (50.0)	0.110
Drainage tube	16 (12.2)	1 (3.0)	15 (15.3)	0.071
Parenteral nutrition	26 (19.8)	4 (12.1)	22 (22.4)	0.198
Nasogastric feeding	122 (93.1)	30 (90.9)	92 (93.9)	0.606
Antibiotic exposure, n (%)				
Penicillins	17 (13.0)	7 (21.2)	10 (10.2)	0.134
Glycopeptides	30 (22.9)	3 (9.1)	27 (27.6)	**0.029**
Oxazolidinones	32 (24.4)	7 (21.2)	25 (25.5)	0.735
Cephalosporins	55 (42.0)	16 (48.5)	39 (39.8)	0.382
β-lactam-β-lactamase inhibitors	90 (68.7)	22 (66.7)	68 (69.4)	0.771
Carbapenems	63 (48.1)	10 (30.3%)	53 (54.1%)	**0.018**
Quinolones	3 (2.3)	1 (3.0)	2 (2.0)	1.000
Tetracyclines	6 (4.6)	2 (6.1)	4 (4.1)	0.641
Macrolides	23 (17.6)	7 (21.2)	16 (16.3)	0.523
Trimethoprim-sulfamethoxazole	1 (0.8)	0 (0.0)	1 (0.1)	1.000
Laboratory detection				
WBC (Χ10^9^/L), M (IQR)	10.6 (6.3)	9.9 (4.0)	11.0 (7.0)	**0.043**
PLT (Χ10^9^/L), ± s	414.5 ± 198.6	439.9 ± 193.8	405.9 ± 200.4	0.398
CRP (mg/L), M (IQR)	7.7 (23.8)	4.7 (15.0)	8.2 (28.0)	0.197
PCT (ng/mL), M (IQR)	0.1 (0.5)	0.1 (0.0)	0.2 (1.0)	**0.045**
ALB (g/L), ± s	36.8 ± 5.7	37.35 ± 4.5	36.6 ± 6.1	0.518
PA (mg/L), M (IQR)	188.0 (124.0)	201.0 (90.0)	186.0 (136.0)	0.410
Aspartate aminotransferase (U/L), M (IQR)	35.0 (28.0)	30.0 (16.0)	37.5 (38.0)	**0.017**
ALT (U/L), M (IQR)	25.0 (25.3)	19.0 (19.0)	26.0 (30.0)	**0.026**
**Mortality**, n (%)	48 (36.6%)	10 (30.3%)	38 (38.8%)	0.382

M, median; IQR, interquartile range; n, number; x̄, mean; s, standard deviation; PICU, pediatric intensive care unit; CVC, central intravenous catheter; WBC, white blood cell; PLT, platelet; CRP, C-reactive protein; PCT, procalcitonin; ALB, albumin; PA, prealbumin; ALT, alanine aminotransferase; Bold values in the P-value column indicated statistical significance.

However, compared with the OXA-23-producing group, the NDM-1-producing group had significantly lower age, a lower proportion of patients with septic shock and indwelling urethral catheters, and less frequently exposed to glycopeptides and carbapenems. Furthermore, serum levels of WBC, PCT, aspartate aminotransferase, and alanine aminotransferase (ALT) were significantly lower in the NDM-1-producing group (*P* < 0.05).

### Risk factors for NDM-1-producing CRAB infection in children

Variance inflation factors (VIFs) were calculated for variables significant at *P* < 0.05 in univariate analysis, including age, septic shock, indwelling urethral catheters, glycopeptides and carbapenems exposure, WBC count, PCT, aspartate aminotransferase, and ALT. All VIFs were below 5, indicating no substantial collinearity. Then, these factors were entered as candidates into the multivariable logistic regression model. Multivariable logistic regression revealed that indwelling urethral catheters (OR = 0.293; *P* = 0.009) and WBC count (OR = 0.896; *P* = 0.039) were inversely associated with NDM-1-producing CRAB infection (**Table 2**). In another multivariable logistic regression model, indwelling urethral catheters (OR = 2.916; *P* = 0.026) and WBC count (OR = 1.116; *P* = 0.041) showed positively associated with OXA-23-producing CRAB infection (**Supplementary Table S2**). The Hosmer–Lemeshow test indicated a good fit for both multivariable logistic regression models (NDM-1 model: *P* = 0.874; OXA-23 model: *P* = 0.593).

**Table 2:** Multivariable logistic regression analysis of factors associated with NDM-1-producing carbapenem-resistant *Acinetobacter baumannii* infection among pediatric patients.

Variables	Multivariable OR (95% CI)	*P-value*
Indwelling urethral catheters	0.293 (0.117-0.731)	**0.009**
WBC count	0.896 (0.807-0.994)	**0.039**

WBC, white blood cell; Bold values in the P-value column indicated statistical significance.

### Whole-genome sequencing data analysis

To infer the phylogenetic relationships among the 131 pediatric CRAB isolates from Guangxi, a SNP-based tree was constructed (**Figure 2**). The analysis revealed that the NDM-1-producing CRAB isolates were evolutionarily distant from the predominant OXA-23-producing strains. To determine whether the WGS data indicate a clonal outbreak or persistent transmission of the NDM-carrying strain within the hospital, the isolation dates of the strain were analyzed. The results revealed that NDM-1-producing CRAB strains were detected between 2022 and 2024, indicating ongoing clonal persistence of this strain within the hospital.

**Figure 2:**
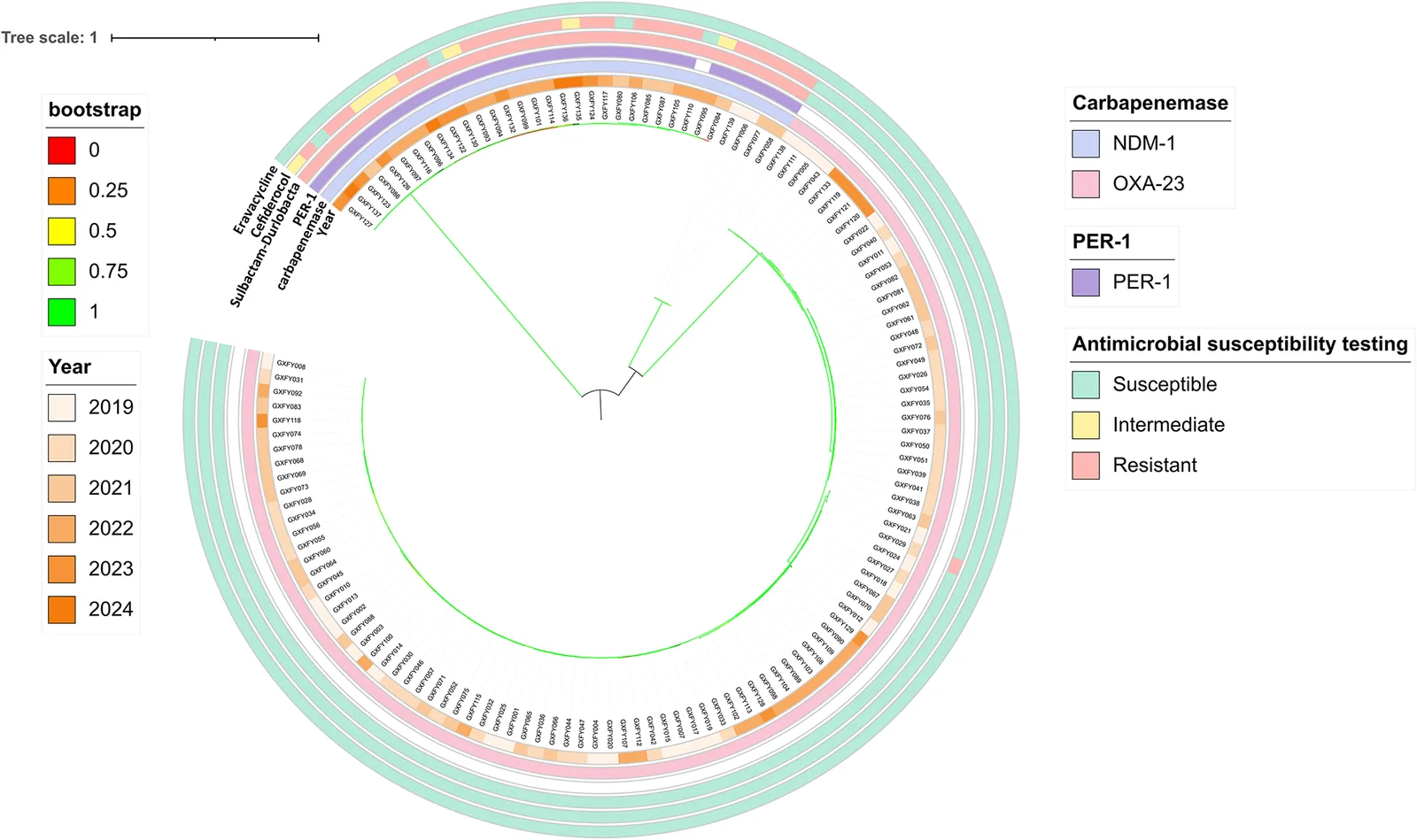
SNP-based phylogenetic tree of 131 carbapenem-resistant *Acinetobacter baumannii* isolates from pediatric patients in Guangxi (2019-2024). The tree is annotated with isolation dates, carbapenemase, PER-1 carriage, and the corresponding antimicrobial susceptibility testing results for sulbactam-durlobactam, cefiderocol, and eravacycline.

The sequencing types, K-locus types (KLs), lipooligosaccharide outer core biosynthesis locus types (OCLs), other antimicrobial resistance genes, and virulence genes of the CRAB strains in this study have been described in our previous study (Liu et al., 2026). We re-emphasized that, among the 33 NDM-1-producing CRAB isolates, 4 belonged to ST718 and 29 to ST1208 according to the Oxford sequence typing scheme. Meanwhile, 88.8% (n=87) of the OXA-23-producing CRAB strains were identified as ST208.

### Nanopore sequencing data analysis

We constructed a circular map of the NDM-1-encoding plasmid from CRAB strain GXFY097 based on Nanopore sequencing data (**Figure 3**). The plasmid was 269,944 bp in length with a GC content of 39.65%. It harbored a T4SS gene cluster, suggesting potential for conjugative transfer. Additionally, it carried multiple antimicrobial resistance genes, including *bla*_NDM-1_ and *bla*_PER-1_.

**Figure 3:**
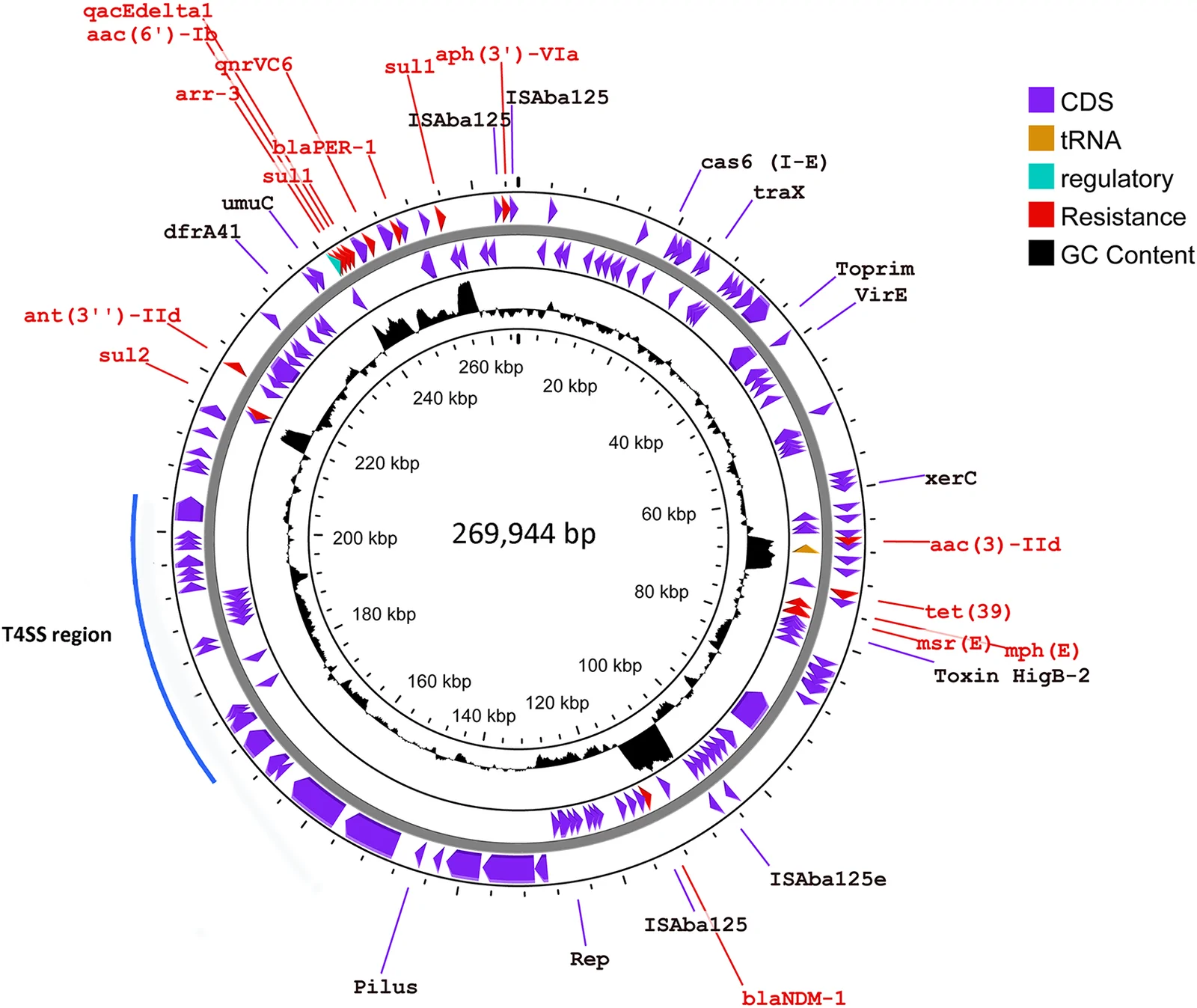
The circular map of the NDM-1-encoding plasmid from CRAB strain GXFY097. The antibiotics resistance genes were highlighted in red.

Based on the short-read mapping data aligned to the NDM-1-encoding plasmid derived from CRAB strain GXFY097, the plasmid was detected in all 32 remaining NDM-1-producing strains, with read coverage ranging from 95% to 99.2% and sequence identity ≥97.0%.

### Antimicrobial susceptibility testing results

The results of AST for the commonly used clinical antibiotics were shown in **Supplementary Table S1**. The resistance rates to piperacillin-tazobactam, levofloxacin, ciprofloxacin, and minocycline in the NDM-1-producing CRAB group were significantly lower than those in the OXA-23-producing CRAB group (*P* < 0.05). However, the resistance rate to cefoperazone-sulbactam was significantly higher in the NDM-1-producing CRAB group (84.8% vs. 18.4%, *P* < 0.001).

In addition, the AST results for sulbactam-durlobactam, cefiderocol, and eravacycline were summarized in **Table 3**. For NDM-1-producing CRAB strains, the MIC ranges against sulbactam-durlobactam, cefiderocol, and eravacycline were 16/4–64/4, 0.5–≥128, and 0.032–0.25 μg/mL, respectively. The MIC50 values were 32/4, 32, and 0.064 μg/mL, respectively, while the MIC90 values were 64/4, ≥128, and 0.064 μg/mL, respectively. For OXA-23-producing CRAB strains, the MIC ranges against sulbactam-durlobactam, cefiderocol, and eravacycline were 0.5/4-16/4, 0.064-0.5, and 0.25-1 μg/mL, respectively. The MIC50 values were 2/4, 0.125, and 0.5 μg/mL, respectively, while the MIC90 values were 2/4, 0.25, and 0.5 μg/mL, respectively. The resistance rate against sulbactam-durlobactam was significantly higher in the NDM-1-producing CRAB group (100.0%) than in the OXA-23-producing CRAB group (1.0%; *P* < 0.001). Moreover, the resistance rate against cefiderocol was significantly higher in the NDM-1-producing CRAB group (66.7%) than in the OXA-23-producing CRAB group (0.0%; *P* < 0.001). In our study, all strains were susceptible to eravacycline.

**Table 3:** Results of antibiotic susceptibility testing for the novel antibiotics in carbapenem-resistant *Acinetobacter baumannii* isolates.

Antibiotics		NDM-1-producing group (n=33)	OXA-23-producing group (n=98)	*P*-value
Sulbactam-Durlobactam (μg/mL)	MIC range	16/4-64/4	0.5/4-16/4	**-**
	MIC50	32/4	2/4	**-**
	MIC90	64/4	2/4	**-**
	Resistant, n (%)	33 (100.0%)	1 (1.0%)	**< 0.001**
Cefiderocol (μg/mL)	MIC range	0.5-≥128	0.064-0.5	–
	MIC50	32	0.125	–
	MIC90	≥128	0.25	–
	Resistant, n (%)	22 (66.7%)	0 (0.0%)	**< 0.001**
Eravacycline (μg/mL)	MIC range	0.032-0.25	0.25-1	–
	MIC50	0.064	0.5	–
	MIC90	0.064	0.5	–
	Resistant, n (%)	0 (0.0%)	0 (0.0%)	1.000

MIC, minimum inhibitory concentration.

MIC50, the minimal drug concentration that inhibits the growth of 50% of isolates.

MIC90, the minimal drug concentration that inhibits the growth of 90% of isolates.

GM, geometric means.

-: Not Applicable.

Bold values in the P-value column indicated statistical significance.

The phenotypic antimicrobial resistance profiles are shown in **Figure 2**. All NDM-1-producing CRAB strains were resistant to sulbactam-durlobactam, while only one OXA-23-producing strain showed resistance. For cefiderocol, 22 of the 33 strains in the NDM-1-producing CRAB group were resistant, while 7 were intermediate. In contrast, all OXA-23-producing CRAB strains were susceptible. In addition, **Figure 4** shows the MIC distribution, for which cefiderocol MIC values ≥128 μg/mL were truncated to 128 μg/mL. The MIC values of OXA-23-producing CRAB strains were lower for sulbactam-durlobactam and cefiderocol compared with NDM-1-producing strains, while slightly higher for eravacycline, despite no strain showing resistance to eravacycline.

**Figure 4:**
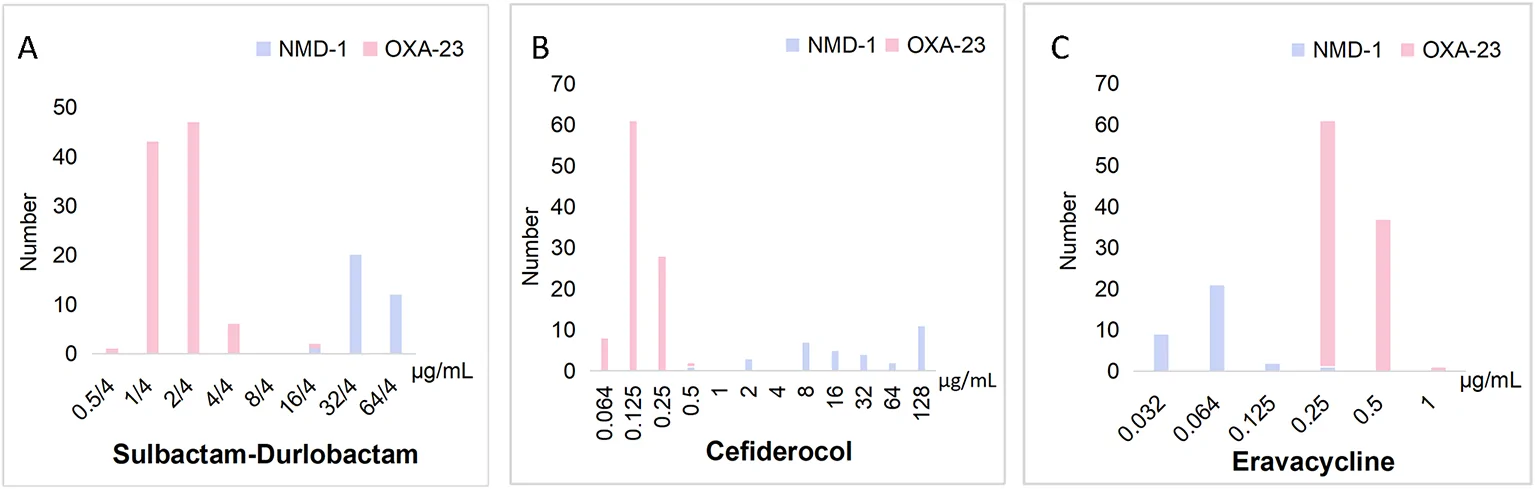
Minimum inhibitory concentration (MIC) distribution of NDM-1- and OXA-23-producing carbapenem-resistant *Acinetobacter baumannii* strains for “**(A)** sulbactam-durlobactam, **(B)** cefiderocol, and **(C)** eravacycline. For cefiderocol, MIC values ≥128 μg/mL were truncated to 128 μg/mL.

### Resistance mechanism analysis

Expect for NDM-1 in resistance of sulbactam-durlobactam and cefiderocol, we analyzed the following resistance mechanism.

PER-1, which is associated with cefiderocol resistance, was detected in 32 of the 33 NDM-1-producing CRAB strains but in none of the OXA-23-producing isolates.

The strain GXFY029, belonging to the OXA-23-producing CRAB group, exhibited resistance to sulbactam-durlobactam. Then, the amino acid sequence of PBP3, encoded by the *ftsI* gene, was compared with that of the reference strain *A. baumannii* ATCC 19606. The comparison revealed no amino acid mutations in PBP3.

No mutations were identified in the ferric uptake receptor genes *pir*A and *piu*A in the cefiderocol-resistant isolates.

## Discussion

CRAB is a globally prevalent nosocomial pathogen that poses a significant threat to infected patients, particularly those who are critically ill and immunocompromised (Karakonstantis et al., 2025). The WHO listed CRAB as a critical priority pathogen in 2024, based on its resistance profile and public health impact (Sati et al., 2025). OXA-23 belongs to class D carbapenemases and functions as a serine hydrolase, whereas NDM-1 is a class B carbapenemase and acts as a metallo-β-lactamase. According to a global study, OXA-23 was the predominant carbapenemase (74.8%) in CRAB, while NDM-1 accounted for only 1.9% (Müller et al., 2023). However, NDM-1 was identified in 25% of isolates in CRAB-infected pediatric patients in Guangxi, China (Liu et al., 2026). NDM-1-positive CRAB, as well as CRAB co-harboring NDM-1 and OXA-23, have been reported to cause an outbreak in Tanta, Egypt (Al-Hassan and Al-Madboly, 2020). A study conducted in India among adult patients found that NDM-positive CRAB isolates exhibited higher resistance rates and were associated with significant mortality (Singh et al., 2024). Given the outbreak potential and high mortality associated with NDM-1-positive CRAB in adults, NDM-1-positive CRAB infections in children warrant special attention and further research.

In this study, we compared the clinical features of NDM-1-producing versus OXA-23-producing CRAB. We found that pediatric patients infected with NDM-1-producing CRAB were younger than those infected with OXA-23-producing CRAB (*P* = 0.045). However, it was different from the study by Sangeeta et al (Singh et al., 2024), in which no significant difference was observed between NDM-positive and non-NDM *A. baumannii* isolates in adults. Therefore, the clinicians should be alert to the presence of NDM-1 in younger children with CRAB infection. This discrepancy suggests that younger children may be more susceptible than adults to NDM-1-producing CRAB infection and its adverse clinical impact. However, mortality rates among pediatric patients with NDM-1-producing CRAB infection were comparable to those with OXA-23-producing CRAB infection. This finding contrasts with the study by Sangeeta et al (Singh et al., 2024), which reported that NDM-positive CRAB isolates were associated with significantly higher mortality. This discrepancy may be attributable to their inclusion of adult patients with *A. baumannii* infection who had multiple comorbidities and received inappropriate empirical therapy.

Furthermore, we identified indwelling urethral catheters and WBC count were negatively associated with NDM-1-producing CRAB infection, while both variables showed positively associated with OXA-23-producing CRAB infection. Of note, the negative coefficients for NDM-1 infection should not be interpreted as true protective effects. Given that the OXA-23 group had a significantly higher proportion of patients with septic shock (21.4% vs. 6.1%), these variables more plausibly function as surrogate markers of greater baseline illness severity and more intensive care exposure in the OXA-23 group, rather than causal determinants of infection type.

The IDSA in its 2026 guidance continued to recommend that sulbactam-durlobactam combined with imipenem or meropenem be used as the preferred treatment for invasive CRAB infections. Sulbactam, a penicillin derivative with a bicyclic β-lactam core and a distinctive sulfone group, is susceptible to hydrolysis by class A, C, and D β-lactamases commonly produced by CRAB (Williams, 1997). Durlobactam, a diazabicyclooctane inhibitor structurally related to avibactam, inhibits class A, C, and D enzymes but lacks activity against class B (McLeod et al., 2024). The combination of durlobactam and sulbactam restores susceptibility in *A. baumannii* strains that produce class A, C, and D β-lactamases (Shapiro et al., 2021). In our study, we found that all NDM-1-producing CRAB strains (100.0%) and one OXA-23-producing CRAB strain (1.0%) were resistant to sulbactam-durlobactam. This resistance profile aligns with the inhibitory spectrum of durlobactam. Therefore, carbapenemase typing is imperative in children with CRAB infection, especially in Guangxi, as different carbapenemase types may dictate entirely distinct therapeutic strategies. Previous studies have identified PBP3 mutations as a common resistance mechanism in OXA-23-producing CRAB resistant to sulbactam-durlobactam (Karruli et al., 2023; Xu et al., 2026). The most frequent mutations S390T, V505L, and T511A identified in stable mutants occurred in the *ftsI* gene, which encodes the sulbactam target PBP3 (Karruli et al., 2023). However, no pbp3 mutations were detected in strain GXFY029 in our study. Other resistance mechanisms, including efflux pump overexpression, may have contributed to this phenotype and warrant further investigation.

Cefiderocol is a siderophore cephalosporin designed for efficient uptake through iron transport channels, leading to a considerable increase in its concentration within the periplasmic space of Gram-negative bacilli (Bilal et al., 2021). The 2026 IDSA guidance recommends cefiderocol, in combination with at least one additional agent—such as high-dose ampicillin-sulbactam, minocycline, or polymyxin B—as an alternative therapeutic option for invasive CRAB infections. As recommended in the report (Stracquadanio et al., 2022), combination therapy may therefore eradicate drug-resistant subpopulations and restore full cefiderocol activity. In our study, the cefiderocol non-susceptibility rate against NDM-1 CRAB was 87.9% (66.7% resistance and 21.2% intermediate), which was substantially higher than the 44.7% reported in a global meta-analysis (Karakonstantis et al., 2024). The substantially higher non-susceptibility rate in our study might be attributed to the co-production of PER-1, which was found in the vast majority (97.0%, 32/33) of our NDM-1-producing CRAB strains. PER- and NDM- β-lactamases are increasingly recognized to mediate cefiderocol resistance in *A. baumannii* (Karruli et al., 2023; Liu et al., 2022; Poirel et al., 2021; Banerjee et al., 2026). Plasmid analysis revealed the co-localization of *bla*_NDM-1_ and *bla*_PER-1_ on the same plasmid, and the resulting co-production of these two β-lactamases may further increase cefiderocol resistance. Plasmid-mediated acquired resistance can be transmitted horizontally, posing a significant challenge to infection control in hospitals. However, 3 NDM-1 CRAB isolates in our study remained susceptible despite harboring *bla*_PER-1_. In a previous study by Poirel et al (Poirel et al., 2021), PER-1-positive *A. baumannii* and *E. coli* isolates exhibited cefiderocol MICs of 2 μg/mL (susceptible), but when exposed to the β-lactamase inhibitor avibactam, the MICs dropped dramatically to ≤0.03 μg/mL. The similar phenomenon was also observed among NDM-1-producing isolates. This indicates that PER-1 and NDM-1 can elevate cefiderocol MICs, but the extent of MIC elevation depends on the level of gene expression or enzymatic activity, which may vary among strains. In isolates with low PER-1 or NDM-1 activity, the MIC may remain below the resistance breakpoint despite the presence of these enzymes. Although cefiderocol resistance acquired during therapy is often linked to iron uptake disruption, we found no *pir*A or *piu*A mutations in our resistant strains. At the time of sample collection (2019-2024), cefiderocol had not yet been approved in China (approval was granted in 2026). Consequently, none of the patients in our cohort had been exposed to this antibiotic, precluding the selective pressure that could drive *pir*A and *pi*uA mutations.

Given these findings, carbapenemase typing is necessary to guide the selection of sulbactam-durlobactam or cefiderocol, and AST is essential for directing the treatment of CRAB infections.

Eravacycline, a synthetic fluorocycline belonging to the tetracycline class, exerts its antibacterial activity by reversibly binding to the bacterial 30S ribosomal subunit (Huang et al., 2024). Consequently, its activity is not affected by β-lactamases. In our study, all (100.0%) NDM-1- and OXA-23-producing CRAB strains were susceptible to eravacycline, a rate higher than the 72.3% susceptibility reported in Zhejiang, China (Zou et al., 2023). Nevertheless, this finding aligns with a multicenter study in China, which reported that the resistance rate of eravacycline against CRAB was less than 5% (Wu et al., 2024). Also, our AST results for eravacycline were consistent with the literature, which generally shows that eravacycline exhibits lower MIC values and more potent bactericidal activity than tigecycline (Zou et al., 2023; Chandran et al., 2023; Wu et al., 2024). We have not analyzed the resistance mechanisms for no eravacycline-resistant CRAB isolates were identified in our study. According to the report, the key resistance mechanisms to eravacycline in clinical isolates include efflux pump upregulation, ribosomal mutations, and plasmid-mediated tet(X) genes (Liang et al., 2026). Furthermore, eravacycline has a broad spectrum of activity, with potent *in vitro* efficacy against numerous Gram-positive and Gram-negative aerobes, anaerobes, and multidrug-resistant organisms (Huang et al., 2024). However, due to its potential to cause tooth discoloration and bone growth inhibition, among other side effects, it should not be used in pregnant women or children below the age of nine (Hoofnagle, 2012). Eravacycline-based therapy may represent an alternative treatment option for older children with infections caused by NDM-1-producing carbapenem-resistant organisms; however, clinical trial evidence is still needed to support this approach.

There are several limitations to this study. First, this was a single-center study with a small sample size. A multi-center investigation with a larger number of cases is needed to improve the reliability and representativeness of the findings. Second, although we performed antimicrobial susceptibility testing for new-generation antibiotics including sulbactam-durlobactam, cefiderocol, and eravacycline, combination susceptibility testing was not conducted. Evaluating antibiotic combinations is particularly important for multidrug-resistant strains and is often needed in clinical settings. Third, our susceptibility testing was limited to *in vitro* assays. Clinical studies are therefore needed to confirm the *in vivo* effectiveness and safety of these agents in pediatric populations.

## Conclusion

NDM-1 has emerged as one of the common carbapenemases in CRAB in pediatric patients in Guangxi, necessitating ongoing surveillance. It conferred resistance to sulbactam-durlobactam and cefiderocol, presenting a major challenge for treatment. Carbapenemase surveillance is critical for the management of CRAB infections in Guangxi, China. Our study provided *in vitro* evidence supporting the susceptibility of CRAB to eravacycline.

## Data Availability

The datasets presented in this study can be found in online repositories. The names of the repository/repositories and accession number(s) can be found in the article/**Supplementary Material**.

## References

[B1] Abdul-Mutakabbir J. C. Alosaimy S. Morrisette T. KebriaeiandM. R. Rybak J. (2020). Cefiderocol: a novel siderophore cephalosporin against multidrug-resistant gram-negative pathogens. Pharmacotherapy 40, 1228–1247. doi: 10.1002/phar.2476 33068441

[B2] Al-Hassan L. L. Al-Madboly L. A. (2020). Molecular characterisation of an Acinetobacter baumannii outbreak. Infect. Prev. Pract. 2, 100040. doi: 10.1016/j.infpip.2020.100040 34368692 PMC8336282

[B3] Alosaimy S. Abdul-Mutakabbir J. C. Kebriaei R. JorgensenandM. S. C. J. Rybak M. J. (2020). Evaluation of eravacycline: a novel fluorocycline. Pharmacotherapy 40, 221–238. doi: 10.1002/phar.2366 31944332

[B4] Asrat H. Samaroo-Campbell J. AtaandJ. S. Quale J. (2023). Contribution of iron-transport systems and β-lactamases to cefiderocol resistance in clinical isolates of Acinetobacter baumannii endemic to New York City. Antimicrob. Agents Chemother. 67, e0023423. doi: 10.1128/aac.00234-23 37212653 PMC10269113

[B5] Banerjee T. Mishra K. Rakshit P. Sharma S. Garg R. Das P. . (2026). Susceptibility of carbapenem-resistant Acinetobacter baumannii and carbapenem-resistant Klebsiella pneumoniae against cefiderocol with reference to their genetic profile in India. Microb. Drug Resist. 32, 111–118. doi: 10.1177/10766294261426703 41783919

[B6] Bartal C. RolstonandL. K. V. I. Nesher L. (2022). Carbapenem-resistant Acinetobacter baumannii: colonization, infection and current treatment options. Infect. Dis. Ther. 11, 683–694. doi: 10.1007/s40121-022-00597-w 35175509 PMC8960525

[B7] Bilal M. El Tabei L. Büsker S. Krauss C. FuhrandM. U. Taubert M. (2021). Clinical pharmacokinetics and pharmacodynamics of cefiderocol. Clin. Pharmacokinet. 60, 1495–1508. doi: 10.1007/s40262-021-01063-5 34420182 PMC8613110

[B8] Boutzoukas A. Doi Y. (2025). The global epidemiology of carbapenem-resistant Acinetobacter baumannii. JAC-Antimicrob. Resist. 7, dlaf134. doi: 10.1093/jacamr/dlaf134 40735512 PMC12305305

[B9] Chandran S. Manokaran Y. Vijayakumar S. Shankar B. A. Bakthavatchalam Y. D. Dwarakanathan H. T. . (2023). Enhanced bacterial killing with a combination of sulbactam/minocycline against dual carbapenemase-producing Acinetobacter baumannii. Eur. J. Clin. Microbiol. Infect. Dis. 42, 645–651. doi: 10.1007/s10096-023-04583-z 36905566

[B10] Hoofnagle J. H. (2012). “ Eravacycline,” in Livertox: Clinical and Research Information on Drug-Induced Liver Injury ( National Institute of Diabetes and Digestive and Kidney Diseases, Bethesda (MD)). 31643176

[B11] Huang P. Y. Hsu C. K. TangandC. H. J. Lai C. C. (2024). Eravacycline: a comprehensive review of *in vitro* activity, clinical efficacy, and real-world applications. Expert Rev. Anti Infect. Ther. 22, 387–398. doi: 10.1080/14787210.2024.2351552 38703093

[B12] Karakonstantis S. Kritsotakis E. I. Tziolos R. N. Vassilopoulou L. Loukaki M. Kypraiou D. . (2025). Mortality due to carbapenem-resistant Acinetobacter baumannii bacteraemia: a 5-year cohort study in intensive care patients. Clin. Microbiol. Infect. 31, 1033–1039. doi: 10.1016/j.cmi.2025.02.018 39978636

[B13] Karakonstantis S. Rousaki M. VassilopoulouandE. L. Kritsotakis I. (2024). Global prevalence of cefiderocol non-susceptibility in Enterobacterales, Pseudomonas aeruginosa, Acinetobacter baumannii, and Stenotrophomonas maltophilia: a systematic review and meta-analysis. Clin. Microbiol. Infect. 30, 178–188. doi: 10.1016/j.cmi.2023.08.029 37666449

[B14] Karruli A. Migliaccio A. Pournaras S. Durante-MangoniandR. E. Zarrilli R. (2023). Cefiderocol and sulbactam-durlobactam against carbapenem-resistant Acinetobacter baumannii. Antibiotics (Basel) 12. doi: 10.3390/antibiotics12121729 38136764 PMC10740486

[B15] Lai C. C. Liu C. Y. Hsueh S. C. Huang Y. T. HsuehandS. P. R. Jean S. S. (2026). Aztreonam-avibactam and cefiderocol against metallo-β-lactamase-producing Enterobacterales and other carbapenem-resistant gram-negative pathogens: microbiological activity and clinical evidence. J. Glob. Antimicrob. Resist. 50, 226–235. doi: 10.1016/j.jgar.2026.06.021 42392447

[B16] Li X. Xie Y. Liu M. Tai C. Sun J. Deng Z. . (2018). oriTfinder: a web-based tool for the identification of origin of transfers in DNA sequences of bacterial mobile genetic elements. Nucleic Acids Res. 46, W229–w234. doi: 10.1093/nar/gky352 29733379 PMC6030822

[B17] Liang D. Yan Y. Wu G. Huang J. Xie M. Tan Y. . (2026). The role of eravacycline in combating multidrug-resistant bacterial infections: insights into efficacy, emerging resistance, and clinical safety. Future Microbiol. 21, 453–464. doi: 10.1080/17460913.2026.2661231 42024584 PMC13166219

[B18] Liu M. Yan C. LiandL. L. Huang L. (2026). Analysis of clinical characteristics and whole-genome sequencing of carbapenem-resistant Acinetobacter baumannii isolates from pediatric patients in Guangxi, China. BMC Microbiol. 26. doi: 10.1186/s12866-026-05188-0 42177429 PMC13378328

[B19] Liu X. Lei T. Yang Y. Zhang L. Liu H. Leptihn S. . (2022). Structural basis of PER-1-mediated cefiderocol resistance and synergistic inhibition of PER-1 by cefiderocol in combination with avibactam or durlobactam in Acinetobacter baumannii. Antimicrob. Agents Chemother. 66, e0082822. doi: 10.1128/aac.00828-22 36377939 PMC9765288

[B20] McLeod S. M. O'Donnell J. P. Narayanan N. MillsandK. J. P. Kaye K. S. (2024). Sulbactam-durlobactam: a β-lactam/β-lactamase inhibitor combination targeting Acinetobacter baumannii. Future Microbiol. 19, 563–576. doi: 10.2217/fmb-2023-0248 38426849 PMC11229585

[B21] Müller C. Reuter S. Wille J. Xanthopoulou K. Stefanik D. Grundmann H. . (2023). A global view on carbapenem-resistant Acinetobacter baumannii. mBio 14, e0226023. doi: 10.1128/mbio.02260-23 37882512 PMC10746149

[B22] Poirel L. SadekandP. M. Nordmann P. (2021). Contribution of PER-type and NDM-type β-lactamases to cefiderocol resistance in Acinetobacter baumannii. Antimicrob. Agents Chemother. 65, e0087721. doi: 10.1128/aac.00877-21 34252309 PMC8448131

[B23] Raro O. H. F. Findlay J. Poirel L. DecousserandP. J. W. Nordmann P. (2026). *In vitro* selection of cefiderocol-resistant mutants in Acinetobacter baumannii harbouring the most common carbapenemase genes. J. Antimicrob. Chemother. 81. doi: 10.1093/jac/dkaf462 41403190 PMC12802952

[B24] Sati H. Carrara E. Savoldi A. Hansen P. Garlasco J. Campagnaro E. . (2025). The WHO Bacterial Priority Pathogens List 2024: a prioritisation study to guide research, development, and public health strategies against antimicrobial resistance. Lancet Infect. Dis. 25, 1033–1043. doi: 10.1016/s1473-3099(25)00118-5 40245910 PMC12367593

[B25] Shapiro A. B. Moussa S. H. McLeod S. M. Durand-RévilleandA. T. Miller A. A. (2021). Durlobactam, a new diazabicyclooctane β-lactamase inhibitor for the treatment of Acinetobacter infections in combination with sulbactam. Front. Microbiol. 12, 709974. doi: 10.3389/fmicb.2021.709974 34349751 PMC8328114

[B26] Singh S. Verma A. Venkatesh V. Verma S. ReddyandA. D. H. Agrawal A. (2024). The clinical impression of NDM-producing Acinetobacter baumannii in intensive care units of the university referral hospital in North India. Indian J. Crit. Care Med. 28, 1044–1049. doi: 10.5005/jp-journals-10071-24834 39882045 PMC11773585

[B27] Smoke S. M. Brophy A. Reveron S. Iovleva A. Kline E. G. Marano M. . (2023). Evolution and transmission of cefiderocol-resistant Acinetobacter baumannii during an outbreak in the burn intensive care unit. Clin. Infect. Dis. 76, e1261–e1265. doi: 10.1093/cid/ciac647 35974429 PMC10169418

[B28] Stracquadanio S. Bonomo C. Marino A. Bongiorno D. Privitera G. F. Bivona D. A. . (2022). Acinetobacter baumannii and cefiderocol, between cidality and adaptability. Microbiol. Spectr. 10, e0234722. doi: 10.1128/spectrum.02347-22 36173300 PMC9603721

[B29] Strateva T. Peykov S. Kalvatchev N. KeuleyanandA. E. Stratev A. (2026). *In vitro* activity of ceftazidime-avibactam, imipenem-cilastatin-relebactam, meropenem-vaborbactam, cefiderocol, and plazomicin against non-fermenting gram-negative isolates obtained from Bulgarian inpatients. Acta Microbiol. Immunol. Hung. 73, 157–167. doi: 10.1556/030.2026.02958 42043870

[B30] Tamma P. D. Heil E. L. Justo J. A. Mathers A. J. SatlinandR. M. J. Bonomo R. A. (2024). Infectious Diseases Society of America 2024 guidance on the treatment of antimicrobial-resistant gram-negative infections. Clin. Infect. Dis. doi: 10.1093/cid/ciae403 39108079

[B31] van den Brand B. Notermans D. W. Verkaik N. J. Lansu S. RossenandA. J. W. A. Hendrickx A. P. A. (2026). CefiderocolFinder: a tool for detecting genetic adaptations implicated in cefiderocol resistance. Clin. Microbiol. Infect. 32, 610–617. doi: 10.1016/j.cmi.2025.12.009 41429344

[B32] Williams J. D. (1997). Beta-lactamase inhibition and *in vitro* activity of sulbactam and sulbactam/cefoperazone. Clin. Infect. Dis. 24, 494–497. doi: 10.1093/clinids/24.3.494 9114205

[B33] Wu Y. Chen J. Zhang G. Li J. Wang T. Kang W. . (2024). In-vitro activities of essential antimicrobial agents including aztreonam/avibactam, eravacycline, colistin and other comparators against carbapenem-resistant bacteria with different carbapenemase genes: a multi-centre study in China 2021. Int. J. Antimicrob. Agents 64, 107341. doi: 10.1016/j.ijantimicag.2024.107341 39304121

[B34] Xu Q. Liu X. Liu H. Yang S. Lei T. Hua X. . (2026). *In vitro* activity and resistance mechanisms of sulbactam/durlobactam against Acinetobacter baumannii clinical isolates in China, (2019-2020). Clin. Microbiol. Infect. 32, 102–109. doi: 10.1016/j.cmi.2025.09.016 41016597

[B35] Zou X. Jin S. Chen L. Li J. Zhang X. Zhou H. . (2023). Antibacterial activity of eravacycline against carbapenem-resistant gram-negative isolates in China: an *in vitro* study. Infect. Drug Resist. 16, 2271–2279. doi: 10.2147/idr.S396910 37090037 PMC10120866

