# Supplementary material for: Clinical characteristics, molecular features and in vitro susceptibility to sulbactam-durlobactam, cefiderocol, and eravacycline in NDM-1-producing versus OXA-23-producing carbapenem-resistant Acinetobacter baumannii isolates from children in Guangxi, China
Source: Front Cell Infect Microbiol. 2026 Oct 8;16:1965770. doi: 10.3389/fcimb.2026.1965770 (PMC13686866; doi:10.3389/fcimb.2026.1965770)
Supplement: Supplementary file 1 [file Table1.docx]

**Table S1. Comparison of Antibiotic Resistance Rates to Traditional Antibiotics in Acinetobacter baumannii Isolates between NDM-1-Producing and OXA-23-Producing Groups**

| **Antibiotics** | **Total (n=131) n (%)** | **NDM-1-producing group (n=33) n (%)** | **OXA-23-producing group (n=98) n (%)** | ***P*-value** |
| --- | --- | --- | --- | --- |
| Ceftazidime | 130 (99.2) | 33 (100) | 97 (99.0) | 1.000 |
| Ceftriaxone | 130 (99.2) | 33 (100) | 97 (99.0) | 1.000 |
| Cefepime | 130 (99.2) | 33 (100) | 97 (99.0) | 1.000 |
| Ampicillin-sulbactam | 123 (93.9) | 33 (100) | 90 (91.8) | 0.201 |
| Ticarcillin-clavulanic acid | 131 (100) | 33 (100) | 98 (100) | / |
| Piperacillin-tazobactam | 123 (93.9) | 28 (84.8) | 95 (96.9) | **0.024** |
| Cefoperazone-sulbactam | 46 (35.1) | 28 (84.8) | 18 (18.4) | **< 0.001** |
| Meropenem | 131 (100) | 33 (100) | 98 (100) | / |
| Imipenem | 125 (95.4) | 31 (93.9) | 94 (95.9) | 0.641 |
| Amikacin | 124 (94.7) | 30 (90.9) | 94 (95.9) | 0.367 |
| Gentamicin | 130 (99.2) | 33 (100) | 97 (99.0) | 1.000 |
| Levofloxacin | 94 (71.8) | 8 (24.2) | 86 (87.8) | **< 0.001** |
| Ciprofloxacin | 101 (77.1) | 13 (39.4) | 88 (89.8) | **< 0.001** |
| Trimethoprim-sulfamethoxazole | 130 (99.2) | 33 (100) | 97 (99.0) | 1.000 |
| Colistin | 1 (0.8) | 0 (0) | 1 (1.0) | 1.000 |
| Minocycline | 41 (31.3) | 3 (9.1) | 38 (38.8) | **0.001** |

/, not applicable
