# Supplementary material for: Clinical characteristics, molecular features and in vitro susceptibility to sulbactam-durlobactam, cefiderocol, and eravacycline in NDM-1-producing versus OXA-23-producing carbapenem-resistant Acinetobacter baumannii isolates from children in Guangxi, China
Source: Front Cell Infect Microbiol. 2026 Oct 8;16:1965770. doi: 10.3389/fcimb.2026.1965770 (PMC13686866; doi:10.3389/fcimb.2026.1965770)
Supplement: Supplementary file 2 [file Table2.docx]

**Table S2. Multivariate logistic regression analysis of factors associated with OXA-23-producing carbapenem-resistant *Acinetobacter baumannii* infection among pediatric patients.**

| **Variables** | **Multivariate**  **OR (95% CI)** | ***P-value*** |
| --- | --- | --- |
| Indwelling urethral catheters | 2.916 (1.137-7.478) | **0.026** |
| WBC | 1.116 (1.004-1.240) | **0.041** |

WBC, white blood cell; Bold values in the P-value column indicated statistical significance.
